# e-Health interventions for healthy aging: a systematic review

**DOI:** 10.1186/s13643-020-01385-8

**Published:** 2020-06-03

**Authors:** Ronald Buyl, Idrissa Beogo, Maaike Fobelets, Carole Deletroz, Philip Van Landuyt, Samantha Dequanter, Ellen Gorus, Anne Bourbonnais, Anik Giguère, Kathleen Lechasseur, Marie-Pierre Gagnon

**Affiliations:** 1 Faculty of Medicine and Pharmacy, Department of Public Health Sciences, Biostatistics and Medical Informatics(BISI) Research Group, Vrije Universiteit Brussel (VUB), Brussels, Belgium; 2 École des sciences infirmières et des études de la santé/School of Nursing and Health Studies, Université de Saint-Boniface, Winnipeg, Manitoba Canada; 3 School of Health Sciences (HESAV), University of Applied Sciences and Arts Western Switzerland, Avenue de Beaumont 21, CH-1011 Lausanne, Switzerland; 4 Faculty of Medicine and Pharmacy, Department of Gerontology, Frailty in Ageing (FRIA) Research Group, Vrije Universiteit Brussel (VUB), Brussels, Belgium; 5 Faculty of Nursing, Université de Montréal, Montreal, Quebec Canada; 6 Research Center of the Institut universitaire de gériatrie de Montréal (CRIUGM), Montreal, Quebec Canada; 7 Faculty of Medicine, Université Laval, Quebec, Canada; 8 Centre de recherche sur les soins et services de première ligne de l’Université Laval (CERSSPL-UL), Quebec, Canada; 9 Research Center of the Centre Hospitalier de Québec-Université Laval (CRCHUQ-UL), Quebec, Canada; 10 Faculty of Nursing Sciences, Université Laval, Québec, 1050 avenue de la Médecine, Quebec, Canada

**Keywords:** e-Health intervention, Healthy aging, e-Health, Information technology

## Abstract

**Background:**

Healthy aging (HA) is a contemporary challenge for population health worldwide. Electronic health (e-Health) interventions have the potential to support empowerment and education of adults aged 50 and over.

**Objectives:**

To summarize evidence on the effectiveness of e-Health interventions on HA and explore how specific e-Health interventions and their characteristics effectively impact HA.

**Methods:**

A systematic review was conducted based on the Cochrane Collaboration methods including any experimental study design published in French, Dutch, Spanish, and English from 2000 to 2018.

**Results:**

Fourteen studies comparing various e-Health interventions to multiple components controls were included. The target population, type of interventions, and outcomes measured were very heterogeneous across studies; thus, a meta-analysis was not possible. However, effect estimates indicate that e-Health interventions could improve physical activity. Positive effects were also found for other healthy behaviors (e.g., healthy eating), psychological outcomes (e.g., memory), and clinical parameters (e.g., blood pressure). Given the low certainty of the evidence related to most outcomes, these results should be interpreted cautiously.

**Conclusions:**

This systematic review found limited evidence supporting the effectiveness of e-Health interventions, although the majority of studies show positive effects of these interventions for improving physical activity in older adults. Thus, better quality evidence is needed regarding the effects of e-Health on the physiological, psychological, and social dimensions of HA.

**Systematic review registration:**

The review protocol was registered in PROSPERO (registration number: CRD42016033163)

## Background

Prospect studies foresee a worldwide growth of people aged over 60 years to at least 2 billion by 2050 [1]. More people are living longer and want to stay active and healthy to fully participate in life. However, decline in the biological, physiological, and cognitive systems inherent to aging may limit full social, cultural, and intellectual engagement in older persons [2]. Therefore, supportive strategies are needed to warrant a good quality of life. Healthy aging (HA) is defined as “the process of optimizing opportunities for physical, social and mental health to enable older people to take an active part in society without discrimination and to enjoy an independent and good quality of life” [3]. HA includes an active engagement with life, optimal cognitive and physical functioning, and low risk of disease that enables older people to participate within their limitations and continue to be physically, cognitively, socially, and spiritually active [4]. Ensuring HA for the population should be a priority in high-income countries today, but also in low-income countries that foresee aging of their population in the near future [5].

Worldwide, baby boomers are reaching the retirement age while policies are levied to keep older adults active in prolonging the working period (i.e., in Greece, France, Denmark) [6–8]. This cohort and onward generations in the “early old age” (defined by the WHO as people aged 50 years or above) [9] increasingly use information and communication technologies (ICT) in their daily activities [10]. With the rapid development of ICTs, which are getting more accessible and easier to use for these older adults, there is a huge potential to develop e-Health interventions targeting the growing population of 50 years and above. The WHO defines e-Health as the electronic exchange of health-related data collected or analyzed through electronic connectivity to improve the efficiency and effectiveness of healthcare delivery [11].

The rise in chronic conditions, which intensify in the last years of life, constitutes a contemporary challenge for health and welfare systems as it has profound implications for the planning and delivery of health and social care. A wide range of literature evidences that a longer life expectancy increases chronic health conditions and pressures the health system in terms of limited resources [12, 13] and public and private spending [14, 15]. Various studies associate steadily spending growth to medical payment schemes [16] or merely to old age with comorbidities [15]. Nevertheless, the World Health Organization (WHO) Brasilia Declaration on Ageing in 1996 stated that “healthy older persons are a resource for their families, their communities and the economy” [17]. In view of these challenges and opportunities, following the United Nations members’ meeting on Aging in Madrid in 2002, the WHO has proposed an active aging policy framework [18] and an age-friendly program plan in 2007 [9].

Among the interventions dedicated to maintain and improve older adults’ active lifestyles and health, those incorporating e-Health receive increasing attention because of their potential to support empowerment and the recognition of their central role in today’s society [19]. There are many examples of successful e-Health applications for health care and health promotion, such as telemedicine, electronic health records, virtual interventions, and personal health monitoring. With respect to HA, e-Health interventions offer older adults the opportunity to access health information and receive health and social care at home. These interactive interventions can empower, engage, and educate older adults [19].

In synthesizing the latest updates, Lattanzio et al. [20] highlight three main domains of development related to advances in technological innovation to support care: (1) disease management, (2) intelligent devices to improve autonomous living and mobility in older persons, and (3) specific needs for active aging. Among common e-Health interventions in support of HA, some are designed for virtual physical exercise [21, 22], and others promote networking [23], an active lifestyle [24], or independence [25]. Interestingly, recent studies contend a high intention to adopt e-Health interventions among older adults [26] and recognize these interventions to be relevant, adapted, and safe to use by these older users [27–30]. Furthermore, e-Health tools are designed to be more portable and lighter [21, 22]. Other authors reported that they offer independence and confidence [27]. Nevertheless, despite technological developments and the multiplication of e-Health applications targeting older adults, knowledge on their effectiveness for supporting HA and its related outcomes has not been synthesized. There is an imperative to determine how e-Health can be used to improve old-age wellbeing.

Technologies that use ubiquitous computing and personalized algorithms play an important role in motivating people to adopt and maintain healthy behaviors as they age [31]. A systematic review of Web 2.0 interventions for chronic disease self-management in older adults found benefits on psychological outcomes as self-efficacy and quality of life, as well as on health behaviors (e.g., physical activity) [32]. Likewise, electronic games could offer huge opportunities for involving older people with cognitive and/or physical disabilities in activities that may support them to participate actively in everyday life [8]. A systematic review found some evidence regarding the effectiveness of exergames, digital gaming systems requiring physical exertion to play the game (e.g., Wii™ games), in improving physical health in older persons [33]. Preschl et al.’s literature review of e-Health interventions targeting depression, anxiety disorders, and dementia in older adults found limited evidence of their effectiveness from high-quality studies, but promising results from smaller studies [34].

To date, to the best of our knowledge, there does not exist a systematic review that addresses the effectiveness of a range of e-Health interventions for supporting HA in all of its dimensions (e.g., physical, social, cognitive). Previous reviews [32–34] provide a starting point for a comprehensive systematic review that could map up current scientific evidence on e-Health interventions for HA.

## Objectives

This systematic review intends to clarify the role of e-Health interventions in promoting HA among older adults. It targets two main objectives: (1) to identify and systematically summarize the best available evidence on the effectiveness of e-Health interventions on outcomes related to HA, as well as adverse effects related to these interventions, and (2) to explore how specific e-Health interventions (e.g., age-friendly, community interventions) and their characteristics (e.g., mode of implementation) may be implemented to effectively impact HA.

## Methods

This systematic review was conducted based on the Cochrane Collaboration methods [35]. The review protocol was registered in PROSPERO (registration number: CRD42016033163). We used the PRISMA checklist [36] (see Supplementary file 1) to ensure reporting of all relevant information related to the systematic review. We also consulted the Synthesis Without Meta-analysis (SWiM) guidelines [37] to guide the use of alternative synthesis methods.

### Types of participants

This review considered studies that include adults aged 50 or more (as 50 years is generally set as the beginning of the young old age) [9], living in the community or in institutional arrangement (e.g., nursing home). Exclusion criteria were as follows: (1) people with terminal illness, or (2) who are hospitalized, or (3) who have severe impaired cognition measured by specific tools such as the Mini Mental State Examination [38].

### Types of interventions

e-Health interventions for healthy aging could include the following: Internet-based interventions, teleconsultations with health care providers, smartphone applications, interactive digital games, electronic records, and information systems. Types of interventions had to correspond to one of the seven technology focus areas proposed by the Center for Technology and Aging [39]. These areas are (1) medication optimization, (2) remote patient monitoring (RPM), (3) assistive technologies, (4) remote training and supervision (RTS), (5) disease management (DM), (6) cognitive fitness and assessment, and (7) social networking. e-Health interventions could take place at home, in a community health center, or another relevant setting. The interventions could be delivered individually or in groups and could take place over one or more sessions of various time frames. We excluded interventions that had an important face-to-face component; interventions that used conventional telephone, television or radio technologies, or technologies without an interactive component; and interventions targeted at treatment or prevention of complications of health problems.

### Types of comparisons

The following comparisons were targeted: (1) any e-Health intervention versus usual service or practice (e.g., any service provided in the health care and/or social system, community, or individual initiative); (2) any e-Health intervention compared to any other e-Health intervention; (3) any e-Health intervention versus any other type of intervention (e.g., intervention with no or only minimal use of ICT); and (4) any e-Health intervention versus no intervention.

### Types of outcomes

This review considered studies that include one or more of the following outcome measures as defined by the “Outcomes of interest to the Cochrane consumers & communication review group” [40]. Primary outcomes related to HA included the following broad categories: (1) quality of life, including life satisfaction, wellbeing, activities of daily living, and leisure activities; (2) health-enhancing lifestyle, including physical activity, healthy diet, and alcohol and tobacco consumption; (3) motivation, including self-efficacy and self-esteem; and (4) social functioning.

Secondary outcomes included (1) knowledge, understanding, and skills acquisition; (2) decision-making including decision made and satisfaction with decision; (3) evaluation of care including goal attainment; (4) social support; and (5) any other behavior related to HA. This study also considered adverse effects related to e-Health interventions in the targeted population. Adverse effects could include social isolation, anxiety, and burden on informal caregivers.

### Types of studies

We considered any experimental study design, including randomized controlled trials, non-randomized controlled trials, and quasi-experimental, before and after studies for inclusion.

Studies published from January 1, 2000, up to April 2018 in English, Dutch, French, or Spanish (languages spoken by team members) were considered for inclusion.

### Search strategy

The search strategy included both published and unpublished studies through a three-step search strategy. An initial exploratory search in Medline and CINAHL was undertaken, as a test, to capture titles and abstracts, with the search equation that comprises common keywords, MeSH term―adapted to each database―and free vocabulary. Then, the results were analyzed to validate and built the final equation. Over this process, an information specialist of the Université Laval (MCL) validated the entire strategy. Finally, the validated search strategy was performed in the following databases: Ovid-Medline®, CINHAL, Cochrane Library, Embase, ERIC, Web of Science, PsycINFO, and Social Work Abstracts (see Supplementary file 2). The search for unpublished studies included clinical trial registers, conference proceedings, and an Internet search on Google and Google Scholar. Thanks to the reference manager Endnote, the research output of references was electronically rid of duplicates. Residual duplicates were manually removed. Finally, the reference list of all identified reports and articles was screened for additional studies.

### Study selection and data extraction

All references were imported in the Rayyan reference screening system [41], and two team members (IB and PVL) independently screened all titles and abstracts for potential inclusion. Their results were combined, and a third reviewer (MPG) solved discrepancies. Thereafter, IB and PVL independently reviewed the full texts of preselected publications for inclusion. RB and MPG checked the list of included and excluded publications and solved discrepancies. IB and PVL performed data extraction independently, using a data extraction form in Excel, which documented details about the study objectives, used interventions, participants, study methods, and outcomes of significance to the review question and specific objectives. The results were compared and completed by RB and MPG.

### Quality appraisal

Because all the selected studies were based on experimental designs, we employed the Cochrane Risk of Bias (ROB) tool for the assessment of possible methodological bias [35]. The reviewers independently rated the quality of each study as either “low,” “unclear,” or “high risk of bias.” They took into consideration the seven domains of the ROB tool:

1. Sequence generation: describes the random components in the sequence generation of the study participants;
2. Allocation concealment: indicates how foreseeable the allocation of participants has proven to be;
3. Blinding of participants: assesses the measures employed to blind the study participants and personnel from knowing the intervention a participant would receive;
4. Blinding of outcome assessment: assesses whether the outcome assessors were blinded from knowing the intervention a participant would receive;
5. Incomplete outcome data: assesses whether the study participants withdrew from the analysis;
6. Selective outcome reporting: assesses a possible selection in expected or pre-specified outcomes, deriving from a systematic difference between reported and nonreported findings, based on the existence of a trial protocol and whether the expected outcomes have been reported in a pre-specified way.
7. Other sources of bias: includes the sample size and the power calculations of the trial that are based on the reported outcome or confounding.

### Data analysis and synthesis

As the populations, interventions, and outcomes described in the included studies were heterogeneous, we were unable to pool quantitative data and conduct a meta-analysis. Therefore, we followed the guidelines regarding alternative forms for combining results [37].

## Results

**Fig. 1:**
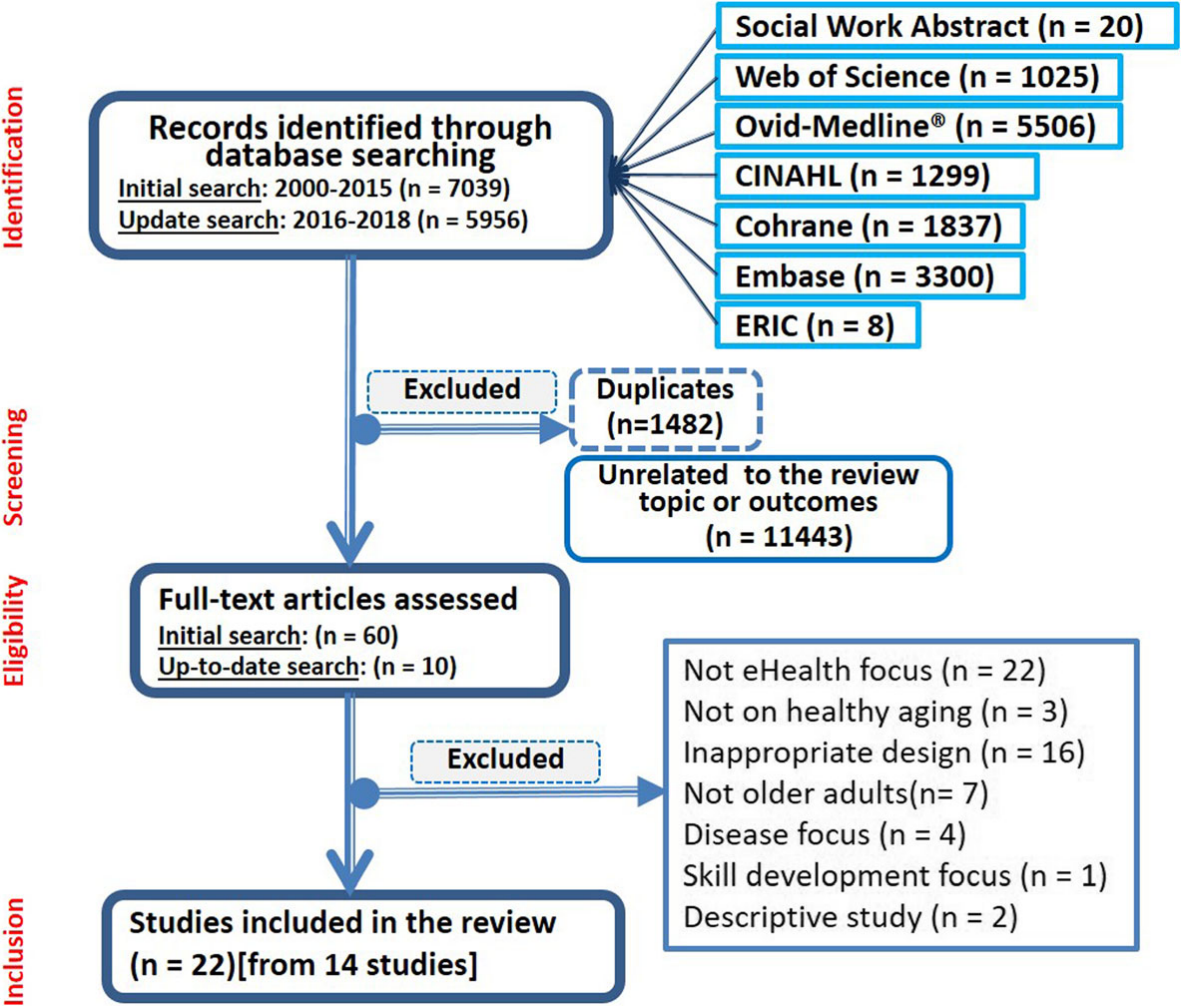
PRISMA flow diagram illustrating the search strategy

Figure 1 shows the study selection flow diagram. The initial search led to 7039 potentially relevant citations. After screening titles and abstracts, 60 publications were kept for further assessment, of which 10 articles were finally retained for the review [24, 42–50]. An updated search ran in May 2018 resulted in the inclusion of three additional peer-review publications [51–53] and one thesis [54].

In total, 21 peer-review articles and one thesis—stemmed from 14 studies—were finally retained. The list of excluded publications and reasons for exclusion is provided in Appendix (Supplementary file 3).

The characteristics of the 14 included studies are presented in Table 1. Five studies were conducted in the USA [24, 43–45, 54], one in Japan [52], and the remaining eight in Europe [42, 46–51]. Among European studies, three were conducted in the Netherlands [48, 49, 51], two in the UK [47, 53], and one in three countries (Greece, Spain, and Sweden) [42]. Furthermore, three studies presented different parts of their results in distinct publications: (i) Wijsman et al. [51, 62, 63], (ii) van het Reve et al. [50, 60, 61], and (iii) Peels et al. [48, 55–57].

**Table 1:** Characteristics of included studies

Study ID Country Related publications	Study design, duration of the intervention	Population and setting Number of participants (*n*) Age (years) Female/male	Description of the intervention Intervention group (IG) Control group (CG)	Outcome measures (primary outcome, *secondary outcome*)	Key findings	Effect size for physical activity (PA) [95% confidence interval]
Ballesteros 2014 [42] Spain, Sweden, Greece	RCT, 12 months	Communities in Spain, Sweden, and Greece *n* = 41 Age range 65–85 GI: mean age 74, GC mean age 75 Female/male: IG 16/9 CG 11/5	ICT-mediated social network: AGNES IG: AGNES CG: chat and coffee with the research team	**Wellbeing** (SPF-IL scale)	IG improved affective dimension (*p* < 0.05) at post-test. IG improved affective dimension 8.92 (SD 1.93) and 10.20 (SD 1.44), at pre- and post-test, respectively	No data on PA
Cook 2015 [24] USA	RCT, 3 months	Workers aged 50 years and older *n* = 278 Age range 50 to 68 Female/male: IG 40/98, CG 50/89	Web-based multimedia program (information and guidance) IG: web-based multimedia program CG: waitlist	**Diet change, mild exercise, self-efficacy** *Eating practices, exercise planning, beliefs about aging*	IG performed better on diet change (*p* 0.048), planning healthy eating (*p* 0.03), and mild exercise (*p* 0.01). IG vs CG showed effects on eating practices (*p* 0.03), exercise self-efficacy (*p* 0.03), exercise planning (*p* 0.03), and aging beliefs (*p* 0.01)	Not possible to calculate effect size
Homma 2016 [52] Japan	Pilot RCT, 3 months	Two districts of Kurihara city *n* = 68 Mean age: IG 65.1, GC 67.2 Female/male: IG 22/13, CG 22/11	IG: videophone group (interactive interviews) CG: document group (printed communication) Telemonitoring of health conducted in both groups	**Physical activity, behavioral change self-assessment** (PA and Diet) *Clinical parameters* (body weight, BMI, blood pressure, albumin) *Perceived health condition and improved lifestyle*	Both CG and IG improved average step per day: CG 5046 vs. 5992 (*p* < 0.01), IG 5829 vs. 7324 (*p* < 0.01); between group (*p* = 0.16). IG improved behavioral change for PA (*p* 0.004), diet (*p* 0.002), and lifestyles (*p* < 0.01). IG improved significantly in most clinical parameters such as blood pressure, HbA1c, albumin, BMI. IG perceived higher improvement in health condition and lifestyle (72.7% vs. 97.1% (*p* < 0.01).	0.21 [− 0.28–0.70]
Irvine 2013 [43] USA	RCT, 12 weeks	Sedentary men and women 55 years and over, community *n* = 368 Mean age 60.3 (SD 4.9) Female/male: IG 127/51, CG 129/61	Web-based intervention to promote physical activity IG: web-based intervention CG: no access to website	**Physical activity** *Body mass index* **Quality of life** SF-12 health survey	IG improved on 13 of 14 outcome measures. IG maintained large gains on all 14 outcomes measures after 6 months.	0.28 [0.05–0.51]
Kim 2013 [44] USA	RCT, 6 weeks	African-American community *n* = 46 Mean age: GI 69.3 (SD 7.3), GC 70.5 (SD 7.5) Female/male: IG 21/5, CG 8/2	Text messaging to motivate walking IG: pedometer, walking instructional manual and text messaging CG: without text messaging	**Physical activity** Step count *Perceived activity levels* Leisure, time, exercise, questionnaire (LTEQ)	IG improved steps vs. CG (679 vs. 398; *p* < 0.05), as well as LTEQ score (*p* < 0.05). Both groups increased their LTEQ score at 6 weeks (*p* < 0.001).	0.12 [− 0.63–0.88]
Kurti 2013 [45] USA	Quasi experimental (controlled trial), 2 months	Community members over 50 years in Florida *n* = 12 Mean age 65.5 Female/male: IG 5/1, CG 5/1	Internet-based intervention (successive 5-day blocks) to increase physical activity in sedentary adults IG: monetary consequences CG: no monetary consequences	**Physical activity**	IG and CG reached the 10,000-step goal. IG vs. CG increased steps (182% vs. 108%) and met steps goals (87% vs. 52%).	Not possible to calculate effect size
Lara 2016 [33] UK	RCT, 8 weeks	Workplaces in Northeast England *n* = 75 Mean age 61 (SD 4) Female/male: IG 38/12, CG 19/6	Web-based intervention (LEAP) IG: LEAP CG: use NHS choices website, UK Department of Health	**Physical activity,** **Mediterranean diet** (MD adherence)	Both IG and CG improved outcomes and no significant differences were detected.	Not possible to calculate effect size
Mouton 2015 [46] Belgium	RCT, 4 arms	One municipality in Belgium *n* = 204 Mean age 65 Female/male: IG1 20/13, IG2 27/13, IG3 25/13, CG 23/15	Web-based, center-based or combined physical activity (PA) intervention IG1: web-based intervention IG2: center-based intervention IG3: mixed (center- and web-based) intervention CG: no intervention	**Physical activity** (PA) level *Readiness for PA, awareness of PA* (general), *awareness of PA* (opportunities in municipality)	IG3 improved in PA level (*p* 0.041), readiness for PA (*p* 0.001). IG3 improved on awareness of PA (*p* 0.003) and awareness of PA opportunities in municipality (*p* 0.001).	0.06 No data available to calculate CI
Myhre 2013 USA	RCT, 3 arms, 8 weeks	2 cohorts from retirement communities in Arizona *n* = 41 Mean age 79.4 Female/male: IG1 9/5, IG2 9/4, CG 11/3	Micro-blogging shared with others or kept private IG1: Facebook IG2: online diary CG: waitlist	*Knowledge, letter memory, keep track*	IG1: knowledge, memory task improved at time 2 vs. baseline (*p* < 0.01); keep track slightly improved (*p* < 0.10)	No data on PA
Nyman 2009 [47] UK	RCT, no duration specified	Community in Southampton *n* = 302 Mean age 70.41 (SD 7.07) Female/male 187/115	Website with tailored advice to undertake strength and balance training (SBT) IG: website with tailored advice CG: generic website	*Attitudes to falls-related intervention scale* (AFRIS)	No significant differences in attitudes toward SBT. IG participants indicated that advice was relevant (*p* 0.017) and activities good (*p* 0.047).	No data on PA
Peels 2013a [48] Netherlands Related publications: Golsteijn 2014 [55], Peels 2012 [56], Peels 2013b [57], Peels 2014a [58], 2014b [59]	Cluster-RCT, 5 arms, 1 year	Community members *n* = 1729 Mean age 62 Female/male: IG1 127/51, IG2 144/112, IG3 111/113, IG4 93/100, CG 158/152	Printed or web-based tailored physical activity intervention IG1: printed basic IG2: print-delivered with environmental information IG3: web-based basic IG4: web-based with environmental information CG: No advice	*Process outcomes* (appreciation, understanding of information)	IG1-IG2: printed intervention vs web-based intervention was significantly higher 92.7–98.2% read, 70.1–76.5% kept, and 39.9–56.8% discussed, and better appreciated (6.06–6.91 vs 5.05–6.11 on a scale of 1–10)	0.10 [− 0.04–0.24]
Slegers 2008 [49] Netherland	Feasibility RCT, 4 arms, 12 months	Community in Maastricht *n* = 236 Age range 64–75 Female/male: ?	Computer training and Internet usage IG 1: training and intervention IG 2: training, no intervention CG1: no training, no intervention CG2: not interested (passive control)	**Physical and psychological well-being** (SF-36) **Social well-being** and *social network*	Most outcomes were not significant. IG participants spent more time on learning new things.	0.24 [− 0.14–0.63]
van het Reve 2014 [50] Switzerland Related publications: Silveira 2013 [60, 61]	Preclinical exploratory trial, 12 weeks	2 institutions for older people and 1 organization providing home nursing care for seniors *n* = 44 Mean age (years) 75 (SD 6) Female/male: IG1 8/5, IG2 10/4, CG 10/7	A tablet with ActiveLifestyle IG1: social group with tablet IG2: individual group with tablet GC: brochure group	*Gait performance* (dual-task walking) **Physical performance** Short physical performance battery (SPPB) *Fall efficacy* Fall efficacy scale *i*nternational (FES-I)	IG1 and IG2 improved significantly in single and dual task walking. IG1, IG2, GC showed SPPB improvement (*p* 0.02) between pre- and post-test. Group difference for FES-I between GC and IG1et IG2 (*p* 0.04).	No data on PA
Wijsman 2013 [55] Netherlands Related publications: Vroege 2014 [62], Broekhuizen 2016 [63]	RCT, 3 months	Community in Leiden *n* = 235 Age range 60–70 Mean age: GI 64.7 (SD 3.0), CG 64.9 (SD 2.8) Female/male: IG 47/72, CG 49/67	Internet-based physical activity intervention: Philips DirectLife IG: Philips DirectLife CG: no intervention	**Physical activity** Moderate-to-vigorous physical activity (MVPA) *Metabolic parameters* **Quality of life** (RAND-36)	IG improved PA, weight, waist circumference, insulin and HbA1c (*p* < 0.001), and MVPA (*p* < 0.001). IG improved emotional and mental health (*p* < 0.03) and health change (*p* < 0.01).	0.58 [0.31–0.85]

*CG* control group, *IG1* intervention group 1, *IG2* intervention group 2, *IG3* intervention group 3, *IG4* intervention group 4

**Fig. 2:**
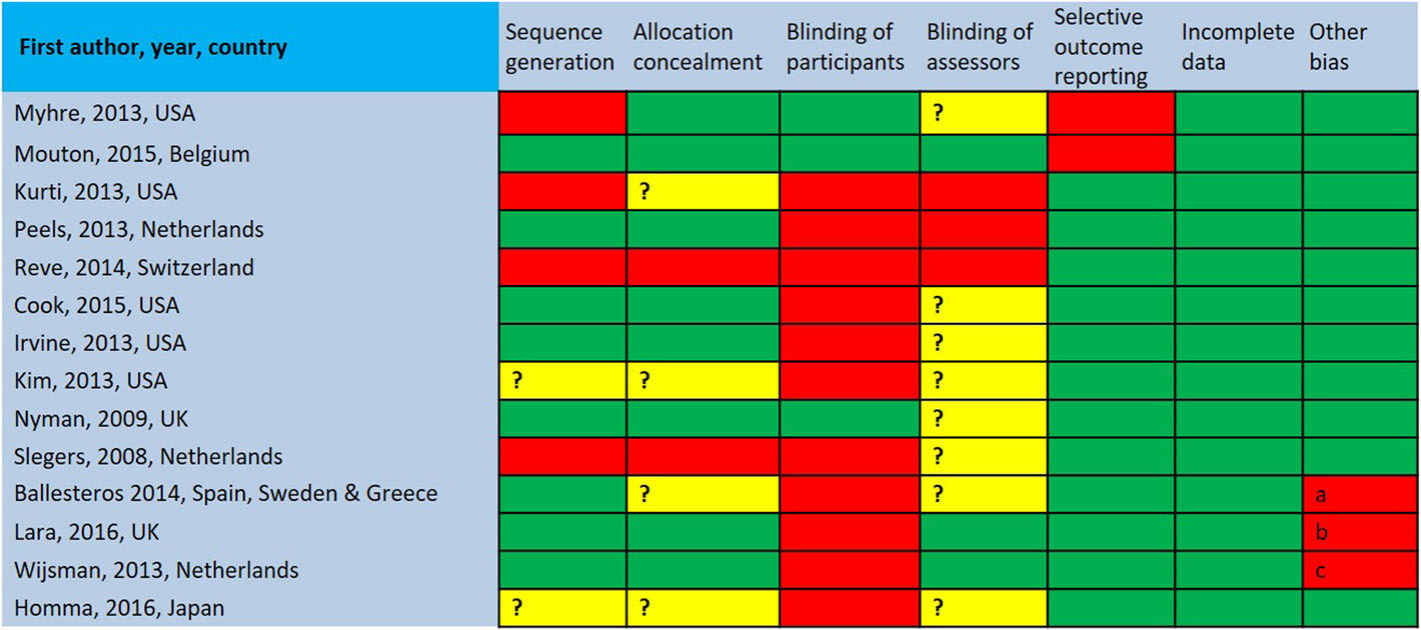
Risk of bias assessment (Other bias: a volunteer, b reporting, c attrition)

When appraising the quality of the retained studies, we first noted that great variation existed with regard to sample size with a minimum of 14 and a maximum of 1729 participants. Seven studies had small samples (*n* < 100)―for a total number of 3645 participants aged between 50 and 88 years old. Also, in the majority of studies, the samples comprised more women than men. Second, as shown in Fig. 2, the risk of bias was moderate to high across the studies, but the source of bias varied. The blinding (participants, personnel, or outcomes assessor) bias was present at a high risk or unclear in most studies. Sequence generation and allocation concealment were variable among the studies, which means that the potential selection biases were foreseeable in half of the studies. Finally, potential risk of bias related to incomplete outcome data and selective outcome was low in a large majority of studies, meaning that there was a low risk of reporting bias. We also noted the use of a wide range of validated questionnaires (such as quality of life: RAND 36; physical and psychological well-being: SF-36; wellbeing: SPF-IL scale) and non-validated rating scales (such as behavioral change self-assessment, IT literacy, engagement in activity) to assess the impact of the interventions.

### Focus area of the technology

All the included study interventions were primarily Internet-based. These interventions were often compared with either paper-based interventions, interventions with a videophone component, mixed intervention, tailored or not. The technology devices that were part of the interventions consisted mainly of computers, tablets, or mobile phones. In reference to the Center for Technology and Aging classification [39], three areas are represented in this systematic review: remote patient monitoring, remote training and supervision, and social networking. One study consisted of an educational program with telemonitoring of step count, blood pressure, and body weight [52]. Most studies aimed to detect, train, and supervise patient remotely. One intervention was personalized with participants’ information provided during the use of the web-based intervention [53], other interventions included information provision to increase daily physical activity [50, 51], or through a Web site with a tailored advice to undertake strength and balance training [47]. Finally, two studies evaluated social networking: one focused on Facebook and the use of an online diary [54], the second on an ICT-mediated social network [42].

As for the remaining studies, Cook et al. [24] focused more widely on health promotion goals (diet, physical activity, stress, tobacco use), whereas Slegers et al. [49] and van het Reve et al. [50] focused on computer training and Internet usage. Lastly, Homma et al. [52] focused on information technology literacy.

With respect to the outcomes, the majority of included studies (11/14) focused on physical activity (PA) [24, 43–48, 50–53] with some focusing on the effect of physical activity on metabolic health and quality of life [51, 52] and another covering increasing healthy behavior [24]. The three other e-Health interventions targeted multiple dimensions including cognitive function, wellbeing, social engagement or connections, quality of life, or lifestyle modification [42, 49, 54].

### Effects of e-Health on healthy behavior outcomes

The most often reported outcome in the included studies was physical activity (PA). Peels et al., comparing paper-based and web-based intervention on PA, concluded that the former was effective in increasing weekly days of sufficient PA (*p* = 0.005) at baseline and 6 months later (*p* = 0.042) [48]. In a similar vein, Irvine et al. showed that a web-based intervention to promote PA improved 13 of the 14 outcome measures and the intervention group maintained large gains on all 14 outcomes measured at 6 months [43]. In the Mouton 2015 study, a mixed intervention (center- and web-based intervention) led to improvement in PA level (*p* = 0.041), readiness for PA (*p* = 0.001), and improved awareness of PA (*p* = 0.003) [46]. In a trial using text messaging, Kim and Glanz contended that motivational text messaging (3 times/week) increases step count (679 vs. 398, *p* < 0.05) as well as perceived activity level (*p* < 0.05) [44]. Using a tablet intervention, van het Reve et al. [50] showed improvement in physical performance for all groups (*p* 0.02) compared to the brochure group in the single and dual-task walking (*p* = 0.03), as well as the falls efficacy (*p* = 0.04) [50]. Likewise, an Internet-based moderate-to-vigorous PA intervention of Wijsman et al. [51] led to a significant improvement of weight and waist circumference (*p* = 0.001). Finally, Homma et al. [52] reported an improvement in steps per day for both videophone intervention (interactive communication) and document groups (*p* < 0.01).

In a trial testing the addition of a monetary incentive to an Internet intervention, Kurti and Dallery concluded a higher percentage of goals achieved (87%) in the group that received the monetary motivation [45]. Nevertheless, some studies were unable to find any significant difference in the PA outcomes targeted. For instance, Lara et al.’s pilot study showed weak and non-significant differences between both groups for PA [53]. However, we should not conclude in the absence of effect for this intervention, as the study was not sufficiently powered.

### Effects of e-Health on clinical parameter outcomes

The study by Wijsman et al. comparing Internet-based PA intervention versus no intervention concluded to a significant improvement in clinical parameters, including insulin and HbA1c (*p* < 0.001); this is for moderate-to-vigorous PA (*p* = 0.001) [51]. Likewise, Homma et al. found significant improvements for blood pressure, HbA1c, and albumin when comparing the videophone intervention group to the document group [52].

### Effects of e-Health on psychological outcomes

Regarding the psychological outcomes, in the Nyman et al. study [47], receiving a web-based tailored advice led to higher ratings of the advice relevance (*p* = 0.017) and goodness of fit of activities (*p* = 0.047). Besides, Wijsman et al. [51] demonstrated that the Internet-based PA intervention improved the emotional and mental health (*p* = 0.03) and health change (*p* < 0.01) in their measure of quality of life. In the Slegers et al. study, however, using computers and the Internet did not influence quality of life, well-being, and mood, nor the social network of healthy older individuals [49].

For their part, Ballesteros et al. found that an ICT-mediated social network improved the affective dimension of wellbeing in their quality of life scale at post-test (*p* < 0.05) [42]. Similarly, Myhre et al.’s Facebook intervention improved knowledge (*p* < 0.01), as well as the Letter Memory task (*p* < 0.01) [54].

### Effects of e-Health on other outcomes

Cook et al. [24] showed that their web-based multimedia program (information and guidance) had a significant effect on diet behavioral change self-efficacy (*p* = 0.05), planning healthy eating (*p* = 0.03), eating practices (*p* = 0.03), exercise self-efficacy (*p* = 0.03), exercise planning (*p* = 0.03), and aging beliefs (*p* = 0.01). In the Peels et al. study [48], the process outcomes showed that the printed group significantly performed better in reading (92.7–98.2%), keeping (70.1–76.5%), and discussing (39.9–56.8%) the advices received. Furthermore, the printed intervention was better appreciated than the web-based intervention (scores 6.06–6.91 versus 5.05–6.11, respectively, on a scale of 1–10) [48]. Moreover, Homma et al. [52] showed a significant positive change in self-assessment of PA (*p* = 0.004), diet (*p* = 0.002), and lifestyles (*p* = 0.005). Participant satisfaction using IT-related devices was significantly higher in the intervention (videophone) group than in the control group (printed documents) (40% vs 15%).

### Outcome synthesis and assessment of the certainty of the evidence

**Fig. 3:**
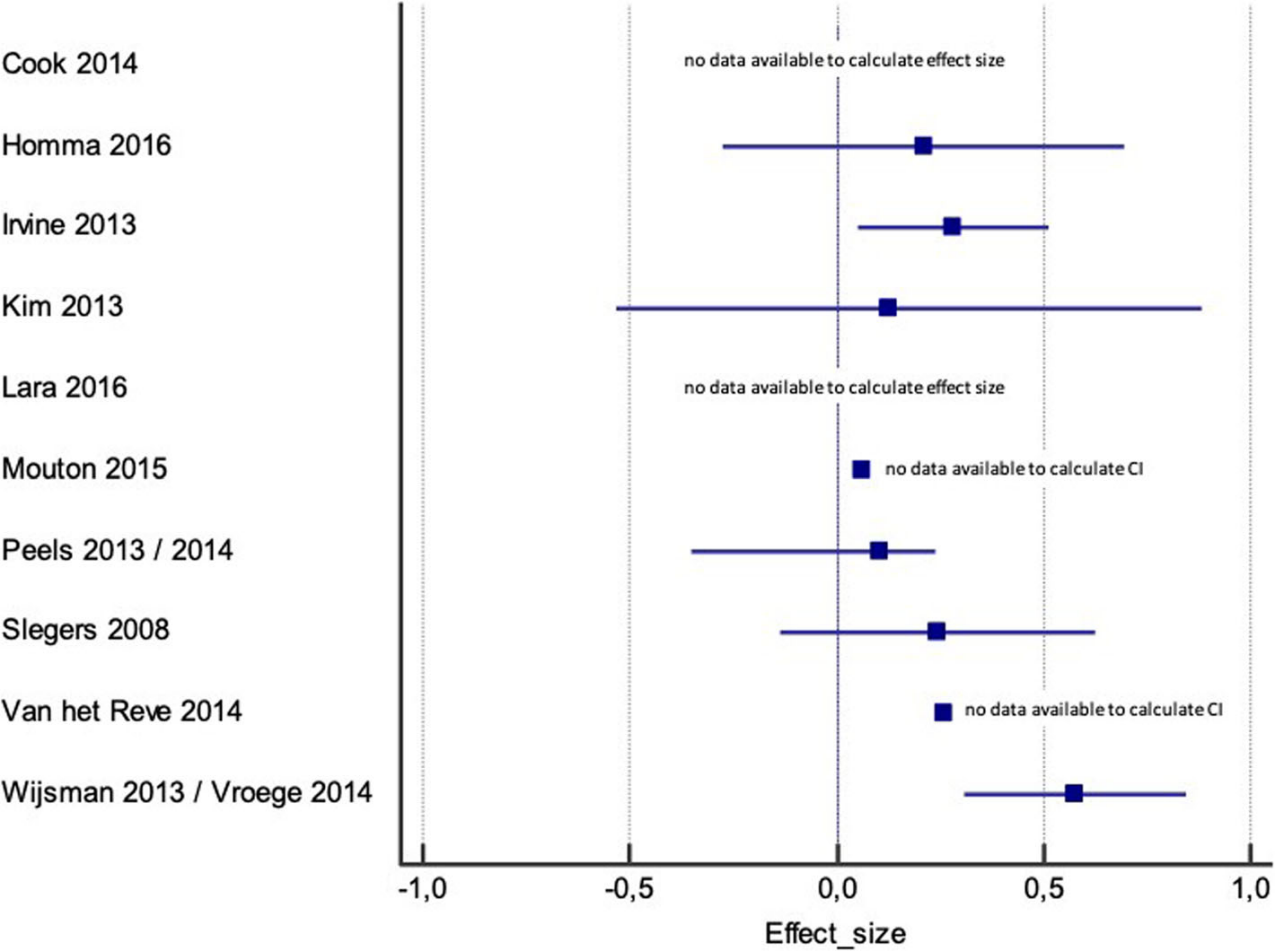
Summary of effect sizes and 95% confidence interval (CI) for physical activity

Due to the important heterogeneity in the studies, it was not possible to conduct a meta-analysis for the outcomes of interest. However, following the SWiM guidance [37], we computed the effect estimates for PA as it was the most frequent outcome reported in the studies. Figure 3 shows the effect size and corresponding 95% confidence interval (CI) for the studies that documented the effectiveness of e-Health on PA. However, some of these studies did not provide sufficient information to calculate the effect size [24, 53] or the CI [46, 50].

We assessed the certainty of the evidence based on the GRADE approach [64] considering the within-trial risk of bias, indirectness, heterogeneity, imprecision, and other considerations (Table 2). As it was not possible to pool the data for most outcomes, we considered the evidence provided by the individual trials as a whole to illustrate the level of evidence for each main category of outcomes. For all outcomes, the certainty of evidence is considered to be very low, mostly due to the risk of bias in individual trials and the imprecision of the estimates given the small sample sizes.

**Table 2:** Certainty of the evidence

Outcome	Number of studies	Risk of bias	Inconsistency	Indirectness	Imprecision	Other considerations	Certainty
Physical activity	11	Serious^a^	Not serious^b^	Serious^d^	Serious^f^	Serious^g^	Very low
Healthy eating	2	Serious^a^	Serious^c^	Serious^d^	Serious^f^	Serious^g^	Very low
Clinical parameters (body mass index, HbAIc, cholesterol)	5	Serious^a^	Serious^c^	Serious^d^	Serious^f^	Serious^g^	Very low
Quality of life	3	Serious^a^	Not serious^b^	Not serious^e^	Serious^f^	Serious^g^	Very low
Cognitive outcomes	2	Serious^a^	Serious^c^	Serious^d^	Serious^f^	Serious^g^	Very low
Psychological outcomes (wellbeing, depression, loneliness)	4	Serious^a^	Serious^c^	Serious^d^	Serious^f^	Serious^g^	Very low
Social outcomes (social support, social functioning)	2	Serious^a^	Serious^c^	Not serious^d^	Serious^f^	Serious^g^	Very low

^a^Most trials had unclear or high risk of bias in one or more domains, among which the lack of blinding of participants, rendering it necessary to downgrade the level of evidence

^b^Although there was some variation in the direction of effect, we did not downgrade the level of evidence since most outcomes showed a positive trend of the effectiveness of eHealth interventions

^c^For these outcomes, there was important heterogeneity in the measures used across studies

^d^Most studies used surrogate outcome measures, among which self-reported measures of physical activity

^e^Quality of life was assessed directly using gold standard measures

^f^When confidence intervals were available, they were usually large. Also, most studies had modest sample sizes

^g^Given the limited number of included studies, we did not compute a funnel plot to check for publication bias, but it is likely that such bias is present given that many studies have a modest sample size

## Discussion

The main objectives of this systematic review were to summarize the best available evidence on the effectiveness of e-Health interventions on HA and to explore how specific e-Health interventions may be implemented to effectively impact HA. To the best of our knowledge, this systematic review is the first to consider the overall effect of e-Health interventions on several dimensions related to healthy aging in older adults.

In this systematic review, we identified a broad variety of interventions that focused on promoting PA and other healthy behaviors, engaging in lifestyle change, and improving physical, psychological, and social wellbeing, which adds to existing literature [65–69]. Overall, most of the included studies were of moderate quality due either to their small sample size, the multiple-component nature of the interventions, their short duration, and the variable quality of the study designs [35].

For healthy older adults, our findings show positive effects of e-Health technology to promote healthy behaviors such as stimulating PA and awareness of PA, to enhance knowledge, and to facilitate behavior change and enhance psychological wellbeing. The use of the Center for Technology and Aging [39] classification system in our work enables the comparison of competing technologies. Furthermore, this classification used in telemedicine and e-Health fields may also facilitate communication among researchers, clinicians, and other users and target the specific technology’s contribution to the health and wellbeing of older adults.

The provision of information was often at the core of the e-Health interventions. Still, there is a need to consider factors related to technology adoption by the older persons, such as interest in learning information and IT literacy [70]. In that line, Vaportzis et al. [71] warned about the following barriers related to IT adoption by older people: lack of instructions and knowledge, health-related barriers, cost, complexity of the technology, lack of social interaction, and communication. Furthermore, Hawley-Hague et al. [72] suggested to consider intrinsic factors related to older adults’ attitudes around control, independence and perceived need/requirements for safety, and their motivation to use and go on using technologies. Several authors also identified some extrinsic factors, including usability, feedback gained, and costs, as important elements supporting older adults’ attitudes and perceptions towards IT use [73, 74].

Other reviews have looked at the impact of ICT use by older people either on physical [75, 76] or on social dimensions [77, 78]. With respect to physical dimensions, the integrative review by Skjaeret et al. [75] found positive effects of exergaming on balance and gait and no major adverse effects. However, the number of included studies was low and most were of limited quality. Nevertheless, PA delivered through e-Health was found to improve adherence to exercise [76].

Chen and Shultz [77] found that ICT use was positively affecting social support, social connectedness, and social isolation among the elderly. However, the effect of ICT on loneliness was inconclusive, with some studies indicating a negative impact. Li et al. [78] reviewed the impact of exergames for older adults on social aspects and found generally positive impacts on loneliness, social connection, and attitudes towards others.

Although the evidence from our synthesis suggests that e-Health interventions are promising, we are still facing several challenges for large-scale implementation of these solutions among older adults. First, there is still a need to strengthen digital health literacy [79, 80]. It is important to recognize that a lack of competence or limitation is often attributed to age-related cognitive decline [81, 82]. Critical competence is needed to effectively evaluate health information [83, 84]. Second, several methodological challenges remain for the evaluation of e-Health intervention. As e-Health interventions are at the intersection of biomedical, behavioral, computing, and engineering research, methods drawn from all these disciplines are required. Experimental designs such as RCTs to evaluate e-Health interventions are cost and time consuming, but remain important for demonstrating their effectiveness and cost-effectiveness [11, 85]. Furthermore, Murray et al. recommend to undertake RCT only after ensuring that the intervention and its delivery package are stable, the intervention can be implemented with high fidelity, and when there is a reasonable likelihood that the overall benefits will be clinically meaningful [86]. Finally, the question of access to Internet arises. The digital divide [87] and the ongoing debate regarding the differences between users and nonusers of online health information among older adults [88], which could reinforce existing social differences, should be considered.

Although it provides a useful synthesis of the current evidence regarding e-Health interventions targeting healthy older adults, this review has some limitations. First, we excluded publications published in languages other than English, Dutch, Spanish, and French. This may have limited the scope of our investigation, but we consider that important international trials were captured by looking at the references of all included studies and searching manually in specialized journals. Second, an important limitation is related to our broad inclusion criteria, which led to include interventions that are quite heterogeneous. Our sample included participants from a wide age range—50 years old and above—thus some interventions might not be applicable to all age groups. This has limited a straightforward comparison and hindered a meta-analysis. However, the use of the Center for Technology and Aging [39] classification helped us to organize the results in a more coherent manner. Based on the types of technologies used and the nature of the interventions, it could be useful to promote a more structured taxonomy to present e-Health interventions in the literature, which could facilitate the identification of relevant studies and the aggregation of their results to inform decisions.

Finally, as the last search was conducted in April 2018, it is possible that more recent trials were not included. We conducted a rapid literature search in PubMed in March 2020 looking for potentially relevant studies published after the last update and identified one published study [89] and six published protocols [90–95], which indicates that several trials of e-Health interventions for HA are currently ongoing. We thus recommend to update this systematic review within the next 2 years as more evidence is likely to change the conclusions of the present systematic review.

## Conclusion

This systematic review contributes to the evidence-base regarding the effectiveness of e-Health interventions in supporting HA. From our perspective, the critical question is how to best shape and direct our efforts to optimize the development and application of these technologies considering older adults’ digital health literacy. As it is an emerging field, the evidence base on e-Health interventions for promoting HA is subject to quick evolution. The pace of technology development is rapid, and the technology could become obsolete at the time the results appear. Thus, innovative evaluation methods are needed to produce high-quality evidence in an appropriate timeframe in order to inform decisions regarding the implementation of effective technologies for HA.

## Supplementary information

**Additional file 1: Supplementary file 1.** PRISMA checklist. **Supplementary file 2.** Search strategies. **Supplementary file 3.** List of excluded references and reasons.

## Data Availability

All data generated or analyzed during this study are included in this published article and its supplementary information files.

## Abbreviations

BMI: Body mass index
CTA: Center for Technology and Aging
DM: Disease management
HA: Healthy aging
ICT: Information and communication technologies
PA: Physical activity
PRISMA: Preferred Reporting Items for Systematic Reviews and Meta-Analyses
RCT: Randomized controlled trial
ROB: Risk of bias
RPM: Remote patient monitoring
RTS: Remote training and supervision
WHO: World Health Organization
