# Supplementary material for: e-Health interventions for healthy aging: a systematic review
Source: Syst Rev. 2020 Jun 3;9:128. doi: 10.1186/s13643-020-01385-8 (PMC7271471; doi:10.1186/s13643-020-01385-8)
Supplement: Supplementary file 1 — Additional file 1: Supplementary file 1. PRISMA checklist. Supplementary file 2. Search strategies. Supplementary file 3. List of excluded references and reasons. [file 13643_2020_1385_MOESM1_ESM.docx]

Supplementary file 1: PRISMA Checklist

| **Section/topic** | **#** | **Checklist item** | **Reported on page #** |
| --- | --- | --- | --- |
| **TITLE** | | |  |
| Title | 1 | Identify the report as a systematic review, meta-analysis, or both. | 1 |
| **ABSTRACT** | | |  |
| Structured summary | 2 | Provide a structured summary including, as applicable: background; objectives; data sources; study eligibility criteria, participants, and interventions; study appraisal and synthesis methods; results; limitations; conclusions and implications of key findings; systematic review registration number. | 2 |
| **INTRODUCTION** | | |  |
| Rationale | 3 | Describe the rationale for the review in the context of what is already known. | 3-4 |
| Objectives | 4 | Provide an explicit statement of questions being addressed with reference to participants, interventions, comparisons, outcomes, and study design (PICOS). | 5 |
| **METHODS** | | |  |
| Protocol and registration | 5 | Indicate if a review protocol exists, if and where it can be accessed (e.g., Web address), and, if available, provide registration information including registration number. | 6 |
| Eligibility criteria | 6 | Specify study characteristics (e.g., PICOS, length of follow-up) and report characteristics (e.g., years considered, language, publication status) used as criteria for eligibility, giving rationale. | 6 |
| Information sources | 7 | Describe all information sources (e.g., databases with dates of coverage, contact with study authors to identify additional studies) in the search and date last searched. | 5-6 |
| Search | 8 | Present full electronic search strategy for at least one database, including any limits used, such that it could be repeated. | 5 |
| Study selection | 9 | State the process for selecting studies (i.e., screening, eligibility, included in systematic review, and, if applicable, included in the meta-analysis). | 6 |
| Data collection process | 10 | Describe method of data extraction from reports (e.g., piloted forms, independently, in duplicate) and any processes for obtaining and confirming data from investigators. | 6-7 |
| Data items | 11 | List and define all variables for which data were sought (e.g., PICOS, funding sources) and any assumptions and simplifications made. | 7 |
| Risk of bias in individual studies | 12 | Describe methods used for assessing risk of bias of individual studies (including specification of whether this was done at the study or outcome level), and how this information is to be used in any data synthesis. | 7 |
| Summary measures | 13 | State the principal summary measures (e.g., risk ratio, difference in means). | N/A |
| Synthesis of results | 14 | Describe the methods of handling data and combining results of studies, if done, including measures of consistency (e.g., I^2^) for each meta-analysis. | 7 |

Page 1 of 2

| **Section/topic** | **#** | **Checklist item** | **Reported on page #** |
| --- | --- | --- | --- |
| Risk of bias across studies | 15 | Specify any assessment of risk of bias that may affect the cumulative evidence (e.g., publication bias, selective reporting within studies). | 17-18 |
| Additional analyses | 16 | Describe methods of additional analyses (e.g., sensitivity or subgroup analyses, meta-regression), if done, indicating which were pre-specified. | N/A |
| **RESULTS** | | |  |
| Study selection | 17 | Give numbers of studies screened, assessed for eligibility, and included in the review, with reasons for exclusions at each stage, ideally with a flow diagram. | 7-8,  Figure 1 |
| Study characteristics | 18 | For each study, present characteristics for which data were extracted (e.g., study size, PICOS, follow-up period) and provide the citations. | 8-9, Supp. File 1 |
| Risk of bias within studies | 19 | Present data on risk of bias of each study and, if available, any outcome level assessment (see item 12). | Not considered |
| Results of individual studies | 20 | For all outcomes considered (benefits or harms), present, for each study: (a) simple summary data for each intervention group (b) effect estimates and confidence intervals, ideally with a forest plot. | 9-15, Supp.File 2 |
| Synthesis of results | 21 | Present results of each meta-analysis done, including confidence intervals and measures of consistency. | N/A |
| Risk of bias across studies | 22 | Present results of any assessment of risk of bias across studies (see Item 15). | 17-18 |
| Additional analysis | 23 | Give results of additional analyses, if done (e.g., sensitivity or subgroup analyses, meta-regression [see Item 16]). | N/A |
| **DISCUSSION** | | |  |
| Summary of evidence | 24 | Summarize the main findings including the strength of evidence for each main outcome; consider their relevance to key groups (e.g., healthcare providers, users, and policy makers). | 15-17 |
| Limitations | 25 | Discuss limitations at study and outcome level (e.g., risk of bias), and at review-level (e.g., incomplete retrieval of identified research, reporting bias). | 17-18 |
| Conclusions | 26 | Provide a general interpretation of the results in the context of other evidence, and implications for future research. | 18 |
| **FUNDING** | | |  |
| Funding | 27 | Describe sources of funding for the systematic review and other support (e.g., supply of data); role of funders for the systematic review. | 19 |

*From:*  Moher D, Liberati A, Tetzlaff J, Altman DG, The PRISMA Group (2009). Preferred Reporting Items for Systematic Reviews and Meta-Analyses: The PRISMA Statement. PLoS Med 6(6): e1000097. doi:10.1371/journal.pmed1000097

For more information, visit: **www.prisma-statement.org**.

Page 2 of 2

Supplementary file 2: Search strategies

PsyInfo

| 1 | ( Title : ( " Health Knowledge" ) OR Abstract : ( " Health Knowledge" ) OR Title : ( " Health Behavior" ) OR Abstract : ( " Health Behavior" ) OR Title : ( " Quality of Life" ) OR Abstract : ( " Quality of Life" ) OR Title : ( " Self-Efficacy" ) OR Abstract : ( " Self-Efficacy" ) OR Title : ( " Social Support" ) OR Abstract : ( " Social Support" ) OR Title : ( " health education" ) OR Abstract : ( " health education" ) OR Title : ( " Life Style" ) OR Abstract : ( " Life Style" ) OR Title : ( " health education" ) OR Abstract : ( " health education" ) OR Title : ( " health knowledge attitude and practice" ) OR Abstract : ( " health knowledge attitude and practice" ) OR Title : ( " health Attitude" ) OR Abstract : ( " health Attitude" ) OR Abstract : ( " health Practice" ) OR Title : ( " health Practice" ) OR Abstract : ( " Health Literacy" ) OR Title : ( " Health Literacy" ) OR Abstract : ( " Health Behaviour" ) OR Title : ( " Health Behaviour" ) OR Abstract : ( " psychco-Social Support" ) OR Title : ( " psychco-Social Support" ) OR Abstract : ( " Risk Reduction Behavior" ) OR Title : ( " Risk Reduction Behavior" ) ) OR ( ( IndexTermsFilt : ( " Health Attitudes" ) OR IndexTermsFilt : ( " Health Behavior" ) OR IndexTermsFilt : ( " Health Education" ) OR IndexTermsFilt : ( " Health Knowledge" ) OR IndexTermsFilt : ( " Health Literacy" ) OR IndexTermsFilt : ( " Health Promotion" ) OR IndexTermsFilt : ( " Quality of Life" ) OR IndexTermsFilt : ( " Quality of Work Life" ) OR IndexTermsFilt : ( " Self Efficacy" ) OR IndexTermsFilt : ( " Self Evaluation" ) OR IndexTermsFilt : ( " Social Support" ) ) ) ) |
| --- | --- |
| 2 | ( " personal digital assistant" ) OR Abstract : ( " personal digital assistant" ) OR Title : ( " medical informatics" ) OR Abstract : ( " medical informatics" ) OR Title : ( " electronic medical record" ) OR Abstract : ( " electronic medical record" ) OR Title : ( " tele consultation" ) OR Abstract : ( " tele consultation" ) OR Title : ( teleconsultation ) OR Abstract : ( teleconsultation ) OR Title : ( " health communication" ) OR Abstract : ( " health communication" ) OR Title : ( " telehealth" ) OR Abstract : ( " telehealth" ) OR Title : ( " interactive digital game" ) OR Abstract : ( " interactive digital game" ) OR Title : ( " electronic information system" ) OR Abstract : ( " electronic information system" ) OR Title : ( " computer-assisted" ) OR Abstract : ( " computer-assisted" ) OR Title : ( " computerized health record" ) OR Abstract : ( " computerized health record" ) OR Title : ( telemedicine ) OR Abstract : ( telemedicine ) OR Title : ( tele-medicine ) OR Abstract : ( tele-medicine ) OR Title : ( telehealth ) OR Abstract : ( telehealth ) OR Title : ( tele-health ) OR Abstract : ( tele-health ) OR Title : ( " mobile health" ) OR Abstract : ( " mobile health" ) OR Title : ( " remote consultation" ) OR Abstract : ( " remote consultation" ) OR Title : ( " electronic health records" ) OR Abstract : ( " personal digital assistant" ) OR Title : ( " public health informatics" ) OR Abstract : ( " public health informatics" ) OR Title : ( " remote monitoring" ) OR Abstract : ( " remote monitoring" ) OR Title : ( " remote monitor" ) OR Abstract : ( " remote monitor" ) OR Title : ( " web based" ) OR Abstract : ( " web based" ) OR Title : ( " web-based" ) OR Abstract : ( " web-based" ) OR Title : ( online ) OR Abstract : ( online ) OR Title : ( on-line ) OR Abstract : ( on-line ) OR Title : ( smartphone ) OR Abstract : ( smartphone ) OR Title : ( " cell phones" ) OR Abstract : ( " cell phones" ) OR Title : ( " information technology personnel" ) OR Abstract : ( " information technology personnel" ) OR Title : ( " occupational health services" ) OR Abstract : ( " occupational health services" ) OR Title : ( internet ) OR Abstract : ( internet ) OR Title : ( " world wide web" ) OR Abstract : ( " world wide web" ) OR Title : ( telenursing ) OR Abstract : ( telenursing ) OR Title : ( tele-nursing ) OR Abstract : ( tele-nursing ) OR Title : ( " health informatics" ) OR Abstract : ( " health informatics" ) OR Title : ( " information technology" ) OR Abstract : ( " information technology" ) OR Title : ( " mobile phone" ) OR Abstract : ( " mobile phone" ) OR Abstract : ( " electronic health records" ) OR Title : ( " personal digital assistant" ) OR IndexTermsFilt : " Telemedicine") OR IndexTermsFilt : ( " Internet" ) OR IndexTermsFilt : ( " Cellular Phones" ) |
| 3 | IndexTermsFilt : ( " Health Education" ) OR ( Title : ( project ) OR Abstract : ( project ) OR Title : ( program ) OR Abstract : ( program ) OR Title : ( programme ) OR Abstract : ( programme ) OR Title : ( intervention ) OR Abstract : ( intervention ) OR Title : ( ' occupational health nursing' ) OR Abstract : ( ' occupational health nursing' ) OR Title : ( ' occupational health service' ) OR Abstract : ( ' occupational health service' ) OR Title : ( ' health education' ) OR Abstract : ( ' health education' ) OR Title : ( ' health promotion' ) OR Abstract : ( ' health promotion' ) OR Title : ( ' preventive health care' ) OR Abstract : ( ' preventive health care' ) OR Title : ( ' primary prevention' ) OR Abstract : ( ' primary prevention' ) OR Title : ( ' health services for the aged' ) OR Abstract : ( ' health services for the aged' ) OR Title : ( support ) OR Abstract : ( support ) |
| 4 | ( Title : ( ' active aging' ) OR Abstract : ( ' active aging' ) OR Title : ( ' active ageing' ) OR Abstract : ( ' active ageing' ) OR Title : ( ' healthy aging' ) OR Abstract : ( ' healthy aging' ) OR Title : ( aging ) OR Abstract : ( aging ) OR Title : ( aged ) OR Abstract : ( aged ) OR Title : ( middle aged ) OR Abstract : ( middle aged ) OR Title : ( old ) OR Abstract : ( old ) OR Title : ( elder* ) OR Abstract : ( elder* ) OR Title : ( senior ) OR Abstract : ( senior ) OR Title : ( ' healthy ageing' ) OR Abstract : ( ' healthy ageing' ) ) |
| 5 | #1 and #2 and #3 and #4 |

Cochrane Library

| 1 | 'healthy aging' or 'healthy ageing' or 'active aging' or 'active ageing' or aging or 'middle aged' or elder* or senior or aged or old:ti,ab,kw (Word variations have been searched) |
| --- | --- |
| 2 | MeSH descriptor: [Aged] explode all trees |
| 3 | MeSH descriptor: [Aging] explode all trees |
| 4 | #1 or #2 or #3 |
| 5 | "health education" or "occupational health service" or "occupational health nursing" or intervention or programme or program:ti,ab,kw or project or support or aged or "health services" or "primary prevention" or "preventive health care" or "health promotion":ti,ab,kw (Word variations have been searched) |
| 6 | MeSH descriptor: [Health Education] explode all trees |
| 7 | MeSH descriptor: [Health Promotion] explode all trees |
| 8 | #5 or #6 or #7 |
| 9 | "information technology personnel" or "cell phones" or smartphone or on-line or online or web-based or "web based" or "web based" or "world wide web" or "remote monitor" or "remote monitoring" or "public health informatics" or "personal digital assistant" or "electronic health records":ti,ab,kw or "remote consultation" or "Cellular Phones" or Tele-medicine or Telemedicine or "mobile phone" or "information technology" or tele-nursing or telenursing or "world wide web" or internet or telehealth or tele-health:ti,ab,kw (Word variations have been searched) |
| 10 | MeSH descriptor: [Telemedicine] explode all trees |
| 11 | MeSH descriptor: [Medical Informatics] explode all trees |
| 12 | MeSH descriptor: [Internet] explode all trees |
| 13 | #9 or #10 or #11 or #12 |
| 14 | 'Health Literacy' or 'health knowledge attitude and practice' or 'health Attitude' or 'health Practice' or 'Health Literacy' or 'Risk Reduction Behavior' or 'Life Style' or 'Social Support' or 'Self-Efficacy' or 'Quality of Life' or 'Health Behavior' or 'Health Knowledge':ti,ab,kw or 'Risk Reduction Behavior' or 'Life Style' or 'Social Support' or 'Self-Efficacy' or 'Quality of Life' or 'Health Behavior' or 'Health Knowledge':ti,ab,kw or 'Risk Reduction Behavior':ti,ab,kw (Word variations have been searched) |
| 15 | MeSH descriptor: [Health Literacy] explode all trees |
| 16 | MeSH descriptor: [Quality of Life] explode all trees |
| 17 | MeSH descriptor: [Life Style] explode all trees |
| 18 | MeSH descriptor: [Social Support] explode all trees |
| 19 | MeSH descriptor: [Self Efficacy] explode all trees |
| 20 | MeSH descriptor: [Health Behavior] explode all trees |
| 21 | MeSH descriptor: [Health Knowledge, Attitudes, Practice] explode all trees |
| 22 | #14 or #15 or #16 or #17 or #18 or #19 or #20#21 |
| 23 | #4 and #8 and #13 and #22 |

ERIC

| S20 | S5 AND S9 AND S15 AND S19 |
| --- | --- |
| S19 | S18 OR S19 OR S20 |
| S18 | TI "Health Behavior" OR AB "Health Behavior" OR TI ("Health Knowledge" OR AB ("Health Knowledge") |
| S17 | TI "Risk Reduction Behavior" OR AB "Risk Reduction Behavior" OR TI "Life Style" OR AB "Life Style" OR TI "Social Support" OR AB "Social Support" OR TI "Self-Efficacy" OR AB "Self-Efficacy" OR TI "Quality of Life" OR AB "Quality of Life" |
| S16 | TI "Health Literacy" OR AB "Health Literacy" OR TI ( "health knowledge attitude and practice" ) OR AB ( "health knowledge attitude and practice" ) OR TI "health Attitude" OR AB "health Attitude" OR TI "health Practice" OR AB "health Practice" OR TI "Health Literacy" OR AB "Health Literacy" OR TI "Health Promotion" OR AB "Health Promotion" |
| S15 | S11 OR S12 OR S13 OR S14 OR S15 |
| S14 | TI telehealth OR AB telehealth OR TI tele-health OR AB tele-health |
| S13 | TI tele-nursing OR AB tele-nursing OR TI telenursing OR AB telenursing OR TI "world wide web" OR AB "world wide web" OR TI internet OR AB internet |
| S12 | TI "remote consultation" OR AB "remote consultation" OR TI "Cellular Phones" OR AB "Cellular Phones" OR TI Tele-medicine OR AB Tele-medicine OR TI Telemedicine OR AB Telemedicine OR TI "mobile phone" OR AB "mobile phone" OR TI "information technology" OR AB "information technology" |
| S11 | TI "web based" OR AB "web based" OR TI "remote monitor" OR AB "remote monitor" OR TI "remote monitoring" OR AB "remote monitoring" OR TI "public health informatics" OR AB "public health informatics" OR TI "personal digital assistant" OR AB "personal digital assistant" OR TI "electronic health records" OR AB "electronic health records" |
| S10 | TI "information technology personnel" OR AB "information technology personnel" OR TI "cell phones" OR AB "cell phones" OR TI smartphone OR AB smartphone OR TI on-line OR AB on-line OR TI online OR AB online OR TI web-based OR AB web-based |
| S9 | S6 OR S7 OR S8 |
| S8 | TI "health promotion" OR AB "health promotion" |
| S7 | TI project OR AB project OR TI support OR AB support OR TI aged OR AB aged OR TI "health services" OR AB "health services" OR TI "primary prevention" OR AB "primary prevention" OR TI "preventive health care" OR AB "preventive health care" |
| S6 | TI "health education" OR AB "health education" OR TI "occupational health service" OR AB "occupational health service" OR TI "occupational health nursing" OR AB "occupational health nursing" OR TI intervention OR AB intervention OR TI programme OR AB programme OR TI program OR AB program |
| S5 | S1 OR S2 OR S3 OR S4 |
| S4 | DE "Adults" OR DE "Older Adults" |
| S3 | DE "Aging (Individuals)" |
| S2 | TI elder* OR AB elder* OR TI senior OR AB senior OR TI aged OR AB aged OR TI "middle aged" OR AB "middle aged" OR TI old OR AB old |
| S1 | TI "healthy aging" OR AB "healthy aging" OR TI "healthy ageing" OR AB "healthy ageing" OR TI "active ageing" OR AB "active ageing" OR TI aging OR AB aging OR TI "active aging" OR AB "active aging" OR TI ageing OR AB ageing |

EMBASE

| #16 | #3 AND #8 AND #12 AND #15 |
| --- | --- |
| #15 | #13 OR #14 |
| #14 | 'health knowledge' OR 'health behavior' OR 'quality of life' OR 'self-efficacy' OR 'social support' OR 'life style' OR 'health knowledge attitude and practice' OR 'health attitude' OR 'health practice' OR 'health literacy' OR 'health behaviour' OR 'psychco-social support' OR 'risk reduction behavior':ab,ti |
| #13 | 'health behavior'/exp OR 'quality of life'/exp OR 'self concept'/exp OR 'risk reduction'/exp |
| #12 | #9 OR #10 OR #11 |
| #11 | 'personal digital assistant'/exp OR 'medical informatics'/exp OR 'electronic medical record'/exp OR 'teleconsultation'/exp OR 'telemedicine'/exp OR 'telehealth'/exp |
| #10 | 'interactive digital game' OR 'electronic information system' OR 'computer-assisted' OR 'computerized health record' OR telemedicine OR 'tele medicine' OR telehealth OR 'tele health' OR 'mobile health' OR 'remote consultation' OR 'electronic health records' OR 'personal digital assistant' OR 'public health informatics':ab,ti OR 'remote monitoring':ab,ti OR 'remote monitor':ab,ti OR 'web based':ab,ti OR 'on line':ab,ti OR 'web based':ab,ti OR smartphone:ab,ti OR 'cell phones':ab,ti OR online:ab,ti OR 'information technology personnel':ab,ti OR 'occupational health services':ab,ti OR internet:ab,ti OR 'world wide web':ab,ti OR 'health communication':ab,ti OR telenursing:ab,ti OR 'tele nursing':ab,ti OR 'health informatics':ab,ti OR 'information technology':ab,ti |
| #9 | 'telenursing'/exp OR 'medical informatics'/exp OR 'information technology'/exp OR 'occupational health service'/exp OR 'internet'/exp OR 'mobile phone'/exp OR 'online system'/exp |
| #8 | #1 OR #2 OR #3 OR #4 OR #5 OR #6 OR #7 |
| #7 | 'primary prevention'/exp |
| #6 | 'health education' OR 'health promotion' OR 'occupational health nursing' OR 'occupational health services' OR 'health services for the aged' OR 'preventive health care' OR 'primary prevention':ab,ti |
| #5 | 'health education'/exp OR 'health promotion'/exp OR 'occupational health nursing'/exp OR 'occupational health service'/exp |
| #4 | project:ab,ti OR program:ab,ti OR support:ab,ti OR programme:ab,ti OR intervention:ab,ti |
| #3 | #1 OR #2 OR #3 OR #4 OR #5 OR #6 OR #7 OR #8 OR #9 OR #11 |
| #2 | senior:ab,ti OR elder*:ab,ti OR old:ab,ti OR aged:ab,ti OR 'middle aged':ab,ti OR 'aged':ab,ti OR 'ageing':ab,ti OR 'aging':ab,ti OR 'active aging' OR 'active ageing' OR 'healthy aging' OR 'healthy ageing':ab,ti |
| #1 | 'aging'/exp OR 'aged'/exp OR 'middle aged'/exp |

Ovid-Medline(R)

| 1 | "active ageing".ab. or "active ageing".ti. or "active aging".ab. or "active aging".ti. or "Healthy ageing".ab. or "Healthy ageing".ti. or "Healthy aging".ab. or "Healthy aging".ti. or Aging.ab. or Aging.ti. Ageing.ab. or Ageing.ti. or Aged.ab. or Aged.ti. or "Middle Aged".ab. or "Middle Aged".ti. or old.ab. or old.ti. or elder*.ab. or elder*.ti. or senior.ab. or senior.ti. |
| --- | --- |
| 2 | exp Aging/ or exp Aged/ or exp Middle Aged/ |
| 3 | 2 or 3 or 4 |
| 6 | Project.ab. or Project.ti. or program.ab. or program.ti. or Support.ab. or Support.ti. or programme.ab. or programme.ti. or intervention.ab. or intervention.ti. or "Health Promotion".ab. or "Health Promotion".ti. |
| 7 | exp Health Promotion/ or |
| 8 | 6 or 7 |
| 10 | exp Occupational Health Nursing/ or exp Occupational Health Services/ or exp Health Services for the Aged/ |
| 11 | "Occupational Health Nursing".ab. or "Occupational Health Nursing".ti. or "Occupational Health Services".ab. or "Occupational Health Services".ti. |
| 12 | "Health Services for the Aged".ab. or "Health Services for the Aged".ti.or "Preventive Health Care".ab. or "Preventive Health Care".ti. or "Primary Prevention".ab. or "Primary Prevention".ti. |
| 13 | Preventive Health Care.mp.or exp Primary Prevention/ |
| 14 | 10 or 11 or 12 or 13 |
| 15 | exp Health Communication/ or exp Telenursing/ |
| 16 | "Health Communication".ab. or "Health Communication".ti. or Telenursing.ab. or Telenursing.ti. or Tele-nursing.ab. or Tele-nursing.ti. or "Health Informatics".ab. or "Health Informatics".ti. or "Information Technology".ab. or "Information Technology".ti. or "Information Technology Personnel".ab. or "Information Technology Personnel".ti. or "Occupational Health Services".ab. or "Occupational Health Services".ti. or "World Wide Web".ab. or "World Wide Web".ti. or Internet.ab. or Internet.ti. or Smartphone.ab. or Smartphone.ti. or online.ab. or online.ti. or on-line.ab. or on-line.ti. or "web based".ab. or "web based".ti. or web-based.ab. or web-based.ti. or webbased.ab. or webbased.ti. or "remote monitor*".ab. or "remote monitor*".ti. or "interactive digital game".af. or "interactive digital game".ti. or "electronic information system".ab. or "electronic information system".ti. or "Computer-Assisted".ab. or "Computer-Assisted".ti. or "Computerized Health Record".af. or "Computerized Health Record".ti. or Telemedicine.ab. or Telemedicine.ti. or Tele-medicine.ab. or Tele-medicine.ti. or Telehealth.ab. or Telehealth.ti. or Tele-health.ab. or Tele-health.ti. or "Mobile health".ab. or "Mobile health".ti. or "Remote Consultation".ab. or "Remote Consultation".ti. or "Mobile health".ab. or "Mobile health".ti. or "Remote Consultation".ab. or "Remote Consultation".ti. or "personal digital assistant".ab. or "personal digital assistant".ti. or "Electronic Health Records".ab. or "Electronic Health Records".ti. or "Public Health Informatics".ab. or "Public Health Informatics".ti. |
| 17 | Health Informatics.mp. or Information Technology.mp. or Information Technology Personnel.mp.or exp Occupational Health Services/ or exp Internet/ or World Wide Web.mp. or Smartphone.mp. or exp Cell Phones/ or Telehealth.mp. or exp Telemedicine/ or Mobile health.mp. or Remote Consultation.mp. or exp Remote Consultation/ or Electronic Health Records.mp. or exp Electronic Health Records/ or Public Health Informatics.mp. or exp Public Health Informatics/ or personal digital assistant.mp. |
| 18 | 15 or 16 or 17 |
| 19 | exp Health Education/ or exp Health Knowledge, Attitudes, Practice/ or exp Health Behavior/ or Health Knowledge.mp. |
| 20 | Quality of Life.mp. or exp "Quality of Life"/ or exp Self Efficacy/ or exp Social Support/ |
| 21 | "Health Knowledge".ab. or "Health Knowledge".ti. or "Health Behavior".ab. or "Health Behavior".ti. or "Quality of Life".ab. or "Quality of Life".ti. or "Self-Efficacy".ab. or "Self-Efficacy".ti. or "Social Support".ab. or "Social Support".ti. or "Life Style".ab. or "Life Style".ti. or "health knowledge attitude and practice".ab. or "health knowledge attitude and practice".ti. or "health Attitude".ab. or "health Attitude".ti. or "health Practice".ab. or "health Practice".ti. or "Health Literacy".ab. or "Health Literacy".ti. or "Health Behaviour".ab. or "Health Behaviour".ti. |
| 22 | "Quality of Life".ab. or "Quality of Life".ti. or "psychco-Social Support".ab. or "psychco-Social Support".ti. or "Risk Reduction Behavior".ab. or "Risk Reduction Behavior".ti. |
| 23 | 19 or 20 or 21 or 22 |

CINAHL

| S13 | **S3 AND S6 AND S9 AND S12** |
| --- | --- |
| S12 | S10 OR S11 |
| S11 | TI ( "health knowledge attitude and practice" ) OR AB ( "health knowledge attitude and practice" ) OR TI "Quality of Life" OR AB "Quality of Life" OR TI "Self Efficacy" OR AB "Self Efficacy" OR TI "psychco-Social Support" OR AB "psychco-Social Support" OR TI "Risk Reduction Behavior" OR AB "Risk Reduction Behavior" OR TI "Life style" OR AB "Life style"  OR TI "Health Knowledge" OR AB "Health Knowledge" OR TI "health Attitude" OR AB "health Attitude" OR TI "health Practice" OR AB "health Practice" OR TI "Health Literacy" OR AB "Health Literacy" OR TI "Health Behavior" OR AB "Health Behavior" OR TI "Health Behaviour" OR AB "Health Behaviour" |
| S10 | (MH "Life Style+") or (MH "Support, Psychosocial+") or (MM "Self-Efficacy") or (MH "Quality of Life+") or (MH "Health Behavior+") or (MM "Health Knowledge") |
| S9 | S7 OR S8 |
| S8 | TI "personal digital assistant" OR AB "personal digital assistant"  OR TI "Electronic Health Records" OR AB "Electronic Health Records" OR TI "Public Health Informatics" OR AB "Public Health Informatics" OR TI "Remote Consultation" OR AB "Remote Consultation" OR TI "Mobile health" OR AB "Mobile health" OR TI Telemedicine OR AB Telemedicine OR TI "Tele-medicine" OR AB "Tele-medicine" OR TI Telehealth OR AB Telehealth OR TI "Tele-health" OR AB "Tele-health" OR TI "Computer-Assisted" OR AB "Computer-Assisted" OR TI "Computerized Health Record" OR AB "Computerized Health Record"  OR TI remote monitor*" OR AB "remote monitor*" OR TI "interactive digital game" OR AB "interactive digital game" OR TI "electronic information system" OR AB "electronic information system" OR TI Smartphone OR AB Smartphone OR TI internet OR AB internet OR TI online OR AB online OR TI on-line OR AB "on-line" OR TI "web based" OR AB "web based" OR TI "web-based" OR AB "web-based" OR TI "Occupational Health Services" OR AB "Occupational Health Services" OR TI "Information Technology" OR AB "Information Technology" OR TI "Information Technology Personnel" OR AB "Information Technology Personnel" OR TI "Health Informatics" OR AB "Health Informatics" OR TI Telenursing OR AB Telenursing OR TI "Tele-nursing" OR AB "Tele-nursing" OR TI webbased OR AB webbased  OR TI "Health Communication" OR AB "Health Communication" |
| S7 | (MM "Remote Consultation")  OR (MH "Telemedicine+") OR (MH "Telehealth+")  OR (MH "Smartphone+") OR (MH "World Wide Web+") OR (MH "Internet+")  OR (MH "Occupational Health Services+")  OR (MM "Information Technology Personnel")  OR MH "Information Technology+")  OR (MH "Health Informatics+") OR (MM "Telenursing") |
| S6 | S4 OR S5 |
| S5 | TI "Primary Prevention" OR AB "Primary Prevention" OR TI "Preventive Health Care" OR AB "Preventive Health Care" OR TI "Health Services for the Aged" OR AB "Health Services for the Aged" OR TI "Occupational Health Services" OR AB "Occupational Health Services" OR TI "Occupational Health Nursing" OR AB "Occupational Health Nursing"  OR  TI "Health Promotion" OR AB "Health Promotion"  OR TI "Health Education" OR AB "Health Education"  OR TI intervention OR AB intervention  OR TI program OR AB program OR TI programme OR AB programme  OR TI Project OR AB Project OR TI program OR AB program OR TI Support OR AB Support |
| S4 | (MH "Preventive Health Care+") OR (MM "Health Services for the Aged")  OR (MH "Occupational Health Services+")  OR (MM "Occupational Health Nursing") OR (MH "Health Promotion+")  OR MH "Health Education+") |
| S3 | S1 OR S2 |
| S2 | TI old OR AB old OR TI elder* OR AB elder* OR TI senior OR AB senior  OR TI Aging OR AB Aging OR TI "Middle Aged" OR AB "Middle Aged" OR TI Aged OR AB Aged OR TI "active ageing" OR AB "active ageing" OR TI "active aging". OR AB "active aging". OR TI "Healthy ageing" OR AB "Healthy ageing" OR TI "Healthy aging" OR AB "Healthy aging" |
| S1 | (MH "Middle Age") or (MH "Aged+") or (MH "Aging+") |

Web of Science

| **#7** | #6 AND #5 AND #4 AND #3 |
| --- | --- |
| **#6** | #6 AND #5 AND #4 AND #3 |
| **#5** | TS=("Health Knowledge" OR "Health Behavior" OR "Quality of Life" OR "Self-Efficacy" OR "Social Support" OR "Life Style" OR "health knowledge attitude and practice" OR "health Attitude" OR "health Practice" OR "Health Literacy" OR "Risk Reduction Behavior" OR "Health Promotion" OR "Health Literacy") |
| **#4** | TS=(internet OR "world wide web" OR telenursing OR tele-nursing OR "health informatics" OR "information technology" OR "mobile phone" OR "electronic health records" OR "personal digital assistant" OR Telemedicine OR Tele-medicine OR "Cellular Phones" OR "remote consultation" OR "electronic health records" OR "personal digital assistant" OR "public health informatics" OR "remote monitoring" OR "remote monitor" OR "web based" OR web-based OR online OR on-line OR smartphone OR "cell phones" OR "information technology personnel" OR "occupational health services") |
| **#3** | TS=("health promotion" OR "preventive health care" OR "primary prevention" OR "health services for the aged" OR support OR project OR program OR programme OR intervention OR "occupational health nursing" OR "occupational health service" OR "health education") |
| **#3** | #1 OR #2 |
| **#2** | TS=(aging OR ageing OR aged OR "middle aged" OR elder* OR old OR senior) |
| **#1** | TS=('active aging' OR 'active ageing' OR 'healthy aging' OR 'healthy aging') |

Social Work Abstracts

| 1 | ('healthy aging' or 'healthy ageing' or 'active aging' or 'active ageing' or aging or 'middle aged' or elder* or senior or aged or old).ti,ab. |
| --- | --- |
| 2 | ("health education" or "occupational health service" or "occupational health nursing" or intervention or programme or program).ti,ab. |
| 3 | (project or support or aged or "health services" or "primary prevention" or "preventive health care").ti,ab. |
| 4 | "health promotion".ti,ab. |
| 5 | #2 or #3 or # 4 |
| 7 | ("information technology personnel" or "cell phones" or smartphone or on-line or online or web-based or "web based" or "web based" or "world wide web" or "remote monitor" or "remote monitoring" or "public health informatics" or "personal digital assistant" or "electronic health records").ti,ab. |
| 8 | ("remote consultation" or "Cellular Phones" or Tele-medicine or Telemedicine or "mobile phone" or "information technology" or tele-nursing or telenursing or "world wide web" or internet).ti,ab. |
| 9 | (telehealth or tele-health).ti,ab. |
| 10 | #7 or #8 or #9 |
| 11 | ((('Health Literacy' or 'health knowledge attitude) and practice') or 'health Attitude' or 'health Practice' or 'Health Literacy' or 'Health Promotion' or 'Risk Reduction Behavior' or 'Life Style' or 'Social Support' or 'Self-Efficacy' or 'Quality of Life' or 'Health Behavior' or 'Health Knowledge').ti,ab. |
| 12 | ('Risk Reduction Behavior' or 'Life Style' or 'Social Support' or 'Self-Efficacy' or 'Quality of Life' or 'Health Behavior' or 'Health Knowledge').ti,ab. |
| 13 | #11 or #12 |
| 14 | #**1 and** #**5 and** #**10 and** #**13** |

Supplementary file 3: List of excluded references and reasons

| Title | Reason of exclusion |
| --- | --- |
| 1. Giesbrecht et al 2016. Clinical benefits of an m health wheelchair skills training program for older adults | Not on eHealth |
| 1. Babiloni & Guarini 2016. Prevention of mental disorders in seniors at risk of Alzheimer's disease in the smart health project: The smartaging platform | Not on eHealth |
| 1. De Cola et al. 2016. Tele-health services for the elderly: A novel southern Italy family needs-oriented model | Wrong design |
| 1. Harries et al. 2016. Effectiveness of a smartphone app in increasing physical activity amongst male adults: a randomised controlled trial | Wrong population |
| 1. Giesbrecht and Miller 2017. A randomized control trial feasibility evaluation of an mHealth intervention for wheelchair skill training among middle-aged and older adults | Not on eHealth |
| 1. David et al. 2017. Combining ICT-based assessment and nonpharmacologic stimulation approaches for the management of frail or cognitively impaired elderly individuals at home | Not on eHealth |
| 1. Jones 2015. Older people going online: its value and before-after evaluation of volunteer support | Wrong design |
| 1. Rodger et al. 2016. Using Education Technology as a Proactive Approach to Healthy Ageing | Wrong population |
| 1. Richard et al., 2016. Healthy Ageing Through Internet Counselling in the Elderly: The HATICE randomised controlled trial for the prevention of cardiovascular disease and cognitive impairment | Ongoing |
| 1. Kim et al 2017. Digital technology to enable aging in place | Not on Healthy Aging |
| 1. Williams et al 2016. Adapting Telemonitoring Technology Use for Older Adults: A Pilot Study | Wrong design |
| 1. Hartzlert al. 2016. Acceptability of a team-based mobile health (mHealth) application for lifestyle self-management in individuals with chronic illnesses | Wrong design |
| 1. De Cocker et al. 2017. What are the working mechanisms of a web-based workplace sitting intervention targeting psychosocial factors and action planning? | Not on Healthy Aging |
| 1. Duncan et al 2016. Balanced: a randomised trial examining the efficacy of two self-monitoring methods for an app-based multi-behaviour intervention to improve physical activity, sitting and sleep in adults | Not on eHealth |
| 1. Gazibara et al. 2016. Computer and online health information literacy among Belgrade citizens aged 66-89 years | Wrong design |
| 1. Taylor et al 2016. Technology support to a telehealth in the home service: Qualitative observations | Not on eHealth |
| 1. Kaiser 2013. Community-partnered health promotion “on the move”: Engaging media to improve the health and quality of life of older adults | Not on eHealth |
| 1. Ts. 2008 E-health for older people: the use of technology in health promotion | Not on eHealth |
| 1. Dickinson 2006. Computer use has no demonstrated impact on the well-being of older adults | Not on eHealth |
| 1. Heo 2011. Exploring the Relationship Between Internet Use and Leisure Satisfaction Among Older Adults | Wrong design |
| 1. James 2013. Internet use and decision making in community-based older adults | Not on eHealth |
| 1. Ji 2010. Older Adults in an Aging Society and Social Computing: A Research Agenda | Not on eHealth |
| 1. Chu 2009. "Partnering with Seniors for Better Health'': computer use and Internet health information retrieval among older adults in a low socioeconomic community | Not on eHealth |
| 1. Cotton 2013. Impact of Internet Use on Loneliness and Contact with Others Among Older Adults: Cross-Sectional Analysis | Wrong design |
| 1. Haris 2014. The Role Of Social Media in Supporting Elderly Quality Daily Life | Wrong design |
| 1. Heo 2015. Exploring the Relationship Between Internet Use and Leisure Satisfaction Among Older Adults | Not on eHealth |
| 1. McComish 2012. Computer and Internet engagement for older adults | Not on eHealth |
| 1. Siriaraya 2014. Exploring the potential of virtual worlds in engaging older people and supporting healthy aging | Wrong design |
| 1. Willner 2015. eHealth 2015 Special Issue: Effects of an assistance service on the quality of life of elderly users | Not on eHealth |
| 1. Knight 2014. Prescribing physical activity for healthy aging: longitudinal follow-up and mixed method analysis of a primary care intervention | Not on eHealth |
| 1. Katsamanis,2006 Computer use and predictors of life satisfaction among older adult computer users | Not on eHealth |
| 1. Zgibor et al 2016. Partnership Building and Implementation of an Integrated Healthy-Aging Program | Not on eHealth |
| 1. Etchemendy 2011. An e-Health platform for the elderly population: The butler system | Wrong design |
| 1. King 2013. Harnessing different motivational frames via mobile phones to promote daily physical activity and reduce sedentary behavior in aging adults | Not on eHealth |
| 1. Thompson 2011. A Holistic approach to assess older adults' wellness using e-Health technologies | Wrong design |
| 1. Gibson 2014. Progress towards Healthy Ageing in Europe: to promote active healthy lifestyles in 45-68 year olds through workplace, rather than traditional health-related settings | Not on eHealth |
| 1. Hall 2012 (editorial) Healthy aging 2.0: the potential of new media and technology | Wrong design |
| 1. Schneider 2015. An online social network to increase walking in dog owners: a randomized trial | Wrong population |
| 1. Hughes 2011 Comparison of two health-promotion programs for older workers | Not on eHealth |
| 1. Schulz 2014. Effects of a web-based tailored multiple-lifestyle intervention for adults: a two-year randomized controlled trial comparing sequential and simultaneous delivery modes | Wrong population |
| 1. Hurling 2007. Using internet and mobile phone technology to deliver an automated physical activity program: Randomized controlled trial | Not on Healthy Aging |
| 1. Maher 2015. A Web-Based, Social Networking Physical Activity Intervention for Insufficiently Active Adults Delivered via Facebook App: Randomized Controlled Trial | Wrong population |
| 1. Foster 2013. Remote and web 2.0 interventions for promoting physical activity | Not on eHealth |
| 1. Safran 2015. The impact of a Web-based app (eBalance) in promoting healthy lifestyles: randomized controlled trial | Not on Healthy Aging |
| 1. De veer 2015. Determinants of the intention to use e-Health by community dwelling older people | Wrong design |
| 1. Balzum 2012. Impact of computer training courses on reduction of loneliness of older people in Finland and Slovenia | Wrong design |
